# Factors affecting the outcome of surgically treated non-iatrogenic traumatic cervical esophageal perforation: 28 years experience at a single center

**DOI:** 10.1186/1749-8090-5-46

**Published:** 2010-05-31

**Authors:** Serdar Onat, Refik Ulku, Kemal M Cigdem, Alper Avci, Cemal Ozcelik

**Affiliations:** 1 Department of Thoracic Surgery, Faculty of Medicine Dicle University, Diyarbakir, Turkey; 2 Department of Pediatric Surgery, Faculty of Medicine Dicle University, Diyarbakir, Turkey; 3 Department of Thoracic Surgery Faculty of Medicine Canakkale Onsekiz Mart University, Canakkale, Turkey

## Abstract

**Background:**

We reviewed our experience with non-iatrogenic traumatic cervical esophageal perforations, paying particular attention to factors affecting the outcome of such cases.

**Methods:**

In total, 30 patients treated surgically between 1980 and 2008 for non-iatrogenic traumatic cervical esophageal perforation in our clinic were reviewed.

**Results:**

There were 25 male and 5 female patients with a median age of 27.5 years. The type of injury was external trauma in 21 (70%) patients and endoluminal injury in the remaining 9 (30%) patients. The mechanism of injury was gunshot in 16 patients, stabbing in 4, falling in 1 (extraluminal injury), and foreign body in 9 (endoluminal injuries). The overall mortality rate was 16.6% (5/30). The mortality rate for extraluminal injuries was 19%, and for endoluminal injuries was 11.1%. Mortality in patients treated within 24 h of sustaining injury was substantially less than in those for whom diagnosis and treatment were delayed (12.5 and 21.4%, respectively). The mortality rate was 33.3% (3/9) for patients with tracheal injuries and 9.5% (2/21) for those without tracheal injuries.

**Conclusions:**

A treatment delay greater than 24 h, the presence of tracheal injury, or extraluminal perforation significantly affected the outcome of surgically treated non iatrogenic traumatic cervical esophageal perforation.

## Introduction

Perforation of the esophagus is a life-threatening condition. This is because the esophagus lacks a serosa and is surrounded by a loose areolar connective tissue, which is unable to prevent the spread of infection and inflammation [1]. Traumatic injuries to the esophagus encompass a heterogeneous group of injuries that may be iatrogenic or non iatrogenic. The cause of injury is ingested foreign body in 12% and trauma in 9% of patients, while iatrogenic injury remains the most frequent cause of esophageal perforation (59%) [2]. Non-iatrogenic traumatic cervical esophageal perforation is a difficult clinical entity, and management requires a thoughtful and individualized approach. The low incidence of this particular problem leads to little clinical experience in surgeons. Thus, we reviewed our experience with non-iatrogenic traumatic esophageal perforation, paying particular attention to factors affecting morbidity and mortality in these patients.

## Methods

The medical records of 30 cervical esophageal perforations due to non-iatrogenic trauma treated surgically in our hospital between 1980 and 2008 were reviewed. Patients in whom instrumentation was clearly and directly responsible for the perforation (even in the presence of a foreign body), patients who died within 24 h due to non-esophageal related complications, patients who suffered from thoracic or intra-abdominal esophageal injury, and patients treated non-operatively for cervical esophageal perforation were excluded. Patients with cervical esophageal perforations due to foreign bodies or with external trauma treated surgically were included.

Demography, clinical presentation, diagnostic investigations, mechanism of injury, associated injuries, time interval between admission and definitive surgical care, management, hospital stay, and morbidity and mortality rates were evaluated. The time interval from occurrence of perforation to its diagnosis and treatment was categorized as early (< 24 h) or late (> 24 h). Esophageal perforation was usually suspected on the basis of presenting signs and symptoms. All patients underwent initial plain neck and chest X-rays. When perforation was suspected, a water-soluble contrast esophagogram was performed. Due to the risk of aspiration pneumonitis, water-soluble esophagograms were not used when tracheo-esophageal fistulas were suspected. In doubtful cases, CT examination with oral contrast was performed.

The type of surgical repair performed (primary repair, drainage alone, primary repair + drainage, primary repair + buttressed by muscle, or exclusion-diversion T-tube) was recorded. Primary repair was performed by a separate two-layer closure of mucosa and muscularis, using interrupted polyglactin sutures. Tracheal repair was performed using single-layer polyglactin sutures.

Postoperative care consisted of nil per os, nasogastric tube replacement, and total parenteral or enteral nutrition. Repetitive radiologic studies were performed when necessary, depending on the patient's fever and clinical condition. A follow-up esophagogram study was done at the end of the fifth postoperative day, based on clinical condition. The same regimen was continued for 10 more days when leakage persisted. The process was repeated until the perforation sealed completely. Multi-slice CT with oral contrast was used primarily for small cervical perforations. Serum C-reactive protein (CRP) and leukocyte counts were used to evaluate the treatment model.

The χ^2 ^test was used for statistical analyses. Where small cell size did not warrant the use of χ^2^, Fisher's exact test was used. Data were also analyzed using Student's t-test and the Mann Whitney U-test. A *p *value < 0.05 was deemed to indicate statistical significance.

## Results

The study included 25 males (83%) and 5 females (17%), with a median age of 27.5 years (range, 2 71). Demographic data of all patients is summarized in Table 1. Of the 30 patients, 21 (70%) suffered from extraluminal injuries, while 9 (30%) had endoluminal perforations, due to foreign bodies. Associated injuries included tracheal injury in nine patients, hemopneumothorax in three, and spinal cord injury in one. All nine patients who had combined tracheal and esophageal injuries suffered from gunshots. Five of these nine patients with combined injuries were explored through a collar incision, while four were explored through an oblique cervical incision. The time interval between esophageal injury and treatment ranged from 4 h to 35 d. The interval between rupture and initial treatment was less than 24 h in 16 patients (53.3%) and longer than 24 h in 14 patients (46.7%).

**Table 1:** Demographic data of all 30 patients, (NS: Non significant)

Factors	Overall (n = 30) **n (%)**	Mortality (n = 5) **n (%)**	P	Morbidity (n = 13) **n (%)**	p	Hospital stay (median day)	P
Sex							
*Male*	25(83.3)	5 (20)	NS	12 (48)	NS	21	
*Female*	5(16.7)	0 (0)		1 (20)		22	
Presentation Time							
*<24 h*	16(53.3)	2 (12.5)	NS	7 (43.8)	NS	15.5	NS
*>24 h*	14(46.7)	3 (21.4)		6 (42.9)		28.5	
Mechanism of injury							
*Gunshot*	16(53.3)	4 (25)	NS	12(75)	0.001	27	NS
*Stab*	4(13.3)	0 (0)		0 (0)		20	
*Fall*	1(3.3)	0 (0)		0 (0)		12	
*Foreign body*	9(30)	1 (11.1)		1 (11.1)		16	
Associated injury							
*Present*	13(43.3)	3 (23.3)	NS	11(85)	0.001	35	0.01
*Absent*	17(56.7)	2 (11.8)		2 (11.8)		15	
Tracheal injury							
*Present*	9 (30)	3 (33.3)	NS	9 (100)	0.001	35	0.01
*Absent*	21 (70)	2 (9.5)		4 (19)		16	
Type of injury							
*Extraluminal*	21(70)	4 (19)	NS	12 (57.1)	NS	26	NS
*Endoluminal*	9 (30)	1 (11.1)		1 (11.1)		16	
Age							
*≤18*	8 (26.6)	1 (12.5)	NS	4 (50)	NS	44	NS
*>18*	22(73.4)	4 (18.2)		9 (41)		21	
Surgical Approach							
*Primary repair*	19(63.3)	2 (10.5)		8 (42.1)	NS	16	NS
*Drainage alone*	8 (26.6)	3 (37.5)	NS	4 (50)	NS	21.5	NS
*Exclusion*	3 (10)	-		1 (33.3)	NS	65	0.04

Pain was the most common presenting symptom (26 patients, 86%). Other symptoms included dysphagia in 20 patients (66%), dysphonia in 10 (33%), and bloody regurgitation in 9 (30%). Subcutaneous emphysema was found on physical examination and appeared radiographically in 23 patients (70%). Two patients had evidence of sepsis preoperatively. Esophageal perforation was confirmed by exploration of the neck in 10 patients (33.3%). The remaining patients were diagnosed by esophagogram (15, 75%) and CT scan (5, 25%). A wide retropharyngeal space was seen on a lateral cervical roentgenogram in three patients (10%) due to abscess formation. Esophagoscopy was performed in patients with perforation due to foreign bodies. These patients had clinical evidence of esophageal injury at the time of presentation. The perforation was missed in three (16.6%) patients by esophagography. In these doubtful cases, CT examination with low-tonicity water-soluble oral contrast confirmed esophageal perforation.

Primary repair was performed in 19 patients. Surgical approaches are listed in Table 1. In 14 patients admitted to our clinic within less than 24 h, primary repair alone was performed. Sternocleidomastoides muscle flaps were used to buttress repairs in five patients (16.7%). Three patients (10%) underwent near total exclusion with cervical T-tube esophagostomy. Three patients required reoperation due to leakage; two underwent redrainage, and one underwent exclusion-T-tube esophagostomy.

Hyperalimentation was administered using total parenteral nutrition (*n *= 24), nasogastric feeding tube (*n *= 3), or feeding jejunostomy and gastrostomy (*n *= 3). At discharge, all patients were on a normal diet without dysphagia. The median hospital stay for all patients ranged from 5 to 99 days (median, 21.5 days). Hospital stays were twice as long in delayed patients, in those younger than 18 years of age, and in those with a gunshot injury, and were significantly longer in patients with additional injuries (*p *< 0.01) and in those surgically treated with exclusion (*p *< 0.04). The median hospital stay for patients with endoluminal perforation (16 days) was shorter than for those with extraluminal perforations (26 days).

Five of the patients required tracheostomies (permanent tracheostomy in one). The overall morbidity rate was 43.3% (13 patients). Complications included sepsis in five, anastomotic leakage in three, wound infection in three, pneumonia in one, and injury of nervus laryngeal recurrentis in one patient. The morbidity rate was significantly higher in patients with gunshot injuries (*p *< 0.001) and tracheal injuries (*p *< 0.001), and was two times higher in male patients.

The overall mortality rate was 16.6% (5 patients; Table 2). The mortality rate in patients with associated tracheal injuries was significantly higher than in those with no tracheal injury. Although not statistically significant, mortality in patients treated within 24 h of sustaining injury was substantially less than in those for whom diagnosis and treatment were delayed. Three patients with delayed treatment died. Of those with gunshot wounds, two were referred to our institution by another hospital. The cause of delayed treatment was delayed presentation in one patient who had ingested a foreign body. The mortality rate was four times higher in patients with gunshot injuries, and three times higher in patients who underwent drainage alone as a surgical treatment. All patients who died had sepsis and multi-organ failure after the development of mediastinitis.

**Table 2:** Features of patients who died

	Etiology	Time interval for treatment	Tracheal injury	Type of treatment
1	Gunshot	> 24 h	Present	Primary suture and drainage
2	Gunshot	< 24 h	Absent	Primary suture
3	Gunshot	< 24 h	Present	Drainage
4	Gunshot	> 24 h	Present	Drainage
5	Foreign body	> 24 h	Absent	Drainage

## Discussion

The most common cause of esophageal perforation is instrumentation; trauma covers only 10% of cases [3,4]. Although traumatic esophageal perforation is rare, its management is a challenge for thoracic surgeons. Penetrating esophageal trauma occurs primarily in the cervical esophagus [5]. Sheely et al. performed a 22-year study involving over 700 patients with penetrating neck trauma, and found 39 patients (5.5%) with cervical esophageal injuries [6]. In our series, 66% of cervical esophageal perforations were due to penetrating cervical injuries. Ingestion of foreign bodies can result in perforation in areas of anatomic narrowing, such as the cricopharyngeus, the impingement of the aortic arch and left main stem bronchus, and the distal esophagus just proximal to the lower esophageal sphincter [2]. In our series, 30% of patients suffered from ingestion of a foreign body.

Diagnosis of esophageal perforation can be missed if the surgeon overlooks its possibility. Plain film findings vary, based on the location and cause of the perforation, as well as on the interval between injury and radiographic examination [7]. Emphysema of the neck is common after cervical perforation and can be detected clinically in 60-95% of patients [7]. We observed emphysema in 70% of our patients. The presence of cervical emphysema is suggestive of a perforated esophagus and warrants further diagnostic study with contrast esophagography. The esophagogram is considered the best method for detecting esophageal perforation. Esophagograms can produce false negative results, at a rate of 10-25% [8]. Of our patients, 60% underwent esophagograms; of these, 16.6% of the results were false negatives. Computed tomographic findings suggestive of esophageal perforation include air in the mediastinal soft tissue surrounding the esophagus, abscessed cavities adjacent to the esophagus, and the presence of an actual communication between air-filled esophagus, and an adjacent mediastinal or paramediastinal air-filled collection [9]. Additionally, CT is particularly useful in follow-up after the initiation of therapy [10]. In our series, CT revealed cervical perforations that had been misdiagnosed by esophagography in three patients. We suggest that CT esophagography is the best diagnostic tool for the evaluation of suspected esophageal perforation. For penetrating trauma, it is very important to establish the trajectory of injury. Diagnosis and treatment was established in 10 of our patients by neck exploration.

Early and prompt surgical intervention remains the mainstay for treatment of cervical esophageal perforation [11,12]. The goal of treatment must be to prevent further soilage from the perforation, to eliminate infection produced by soilage, to restore the integrity and continuity of the gastrointestinal tract, and to restore and maintain adequate nutrition [4]. Primary closure, with or without tissue reinforcement, is the preferable and most frequently used approach for surgical management of esophageal perforations [4]. Primary repair was performed in 63.3% of the patients in our series. Classic teaching cites time from perforation to diagnosis as a predictor of outcome, and various series have identified the first 24 h as an acceptable interval for successful primary repair [13]. Historically, the use of primary repair following a delay in diagnosis of greater than 24 h has been found to be associated with increased mortality [14]. However, some surgeons have recently attempted primary repair, regardless of the interval between the injury and intervention [15-17]. The severe contamination and inflammation that result from delayed treatment of esophageal perforation may preclude primary repair at the time of diagnosis [2]. We advocate drainage and primary suturing, if possible, for late presentations.

Because leakage after suture repair is a frequent occurrence, closure should be supported by a local tissue flap [4]. Sternohyoid, sternothyroid, or sternocleidomastoid muscles may be used to create the flap [3]. We used a sternocleidomastoid muscle flap for cervical perforations in five patients, and all survived.

If the esophageal leak is large and puts the tracheal repair in jeopardy, the salivary stream can be diverted with a "spit fistula," or T-tube. Reconstruction of the esophagus can be done after the tracheal repair has healed [3]. Some investigators have reported successful clinical outcomes with the use of esophageal exclusion and diversion techniques for the treatment of esophageal perforation following late diagnosis and extensive contamination [18]. In our series, three patients were treated using this approach However, this technique should be reserved for those patients in whom other approaches are not feasible [4].

Weiman et al. pointed out that penetrating trauma causes perforations primarily in the cervical esophagus, and morbidity is usually due to associated injuries, such as tracheal injuries [3]. In our series, morbidity and mortality were high in patients who suffered from penetrating injuries. Additionally, mortality in patients who had concomitant tracheal injury was higher than in those without tracheal injury (33.3 and 9.5%, respectively). Associated tracheal injuries also prolonged the duration of hospital stays in our series. The mortality rate for traumatic perforations ranges from 0 to 33% [8,13,15]. Overall, the mortality rate was 16.6% in our series. The mortality rate in patients treated with primary suturing was lower than in those treated with drainage alone (10.5 and 37.5%, respectively). The patients who died after drainage alone were treated in the early 1990 s. The interval from perforation to initiation of treatment is a crucial determinant of outcome after esophageal perforation. Although advances in antibiotic therapy and critical care have reduced complications following delayed diagnosis, a delay in treatment of greater than 24 h is still associated with an increase in mortality [13]. Prompt diagnosis and treatment of esophageal perforation is essential for a favorable outcome. Delay in treatment after esophageal perforation has been found to be associated with higher rates of both complication and mortality [19]. The mortality rate in our patients treated before 24 h was 12.5%, and it was 21.4% for patients treated after 24 h. Attar and colleagues reported that survival decreased from 87 to 55% if treatment was instituted more than 24 h after the onset of symptoms [13]. Nesbitt and Sawyers [20] noted that if treatment was initiated within 24 h, the mortality rate decreased from 56 to 13% over the following years.

In conclusion, although cervical esophageal perforation is a potentially life-threatening condition, early diagnosis and prompt treatment reduce morbidity and mortality. Esophageal injury should be suspected when a gunshot or stab wound is transcervical. Operative therapy remains the mainstay of treatment; however, conservative management may be preferred for certain well-defined situations, and an esophageal T-tube may be used for late perforations. Primary repair of the perforation site and drainage offers the highest probability of survival. Clinical outcome of these patients is influenced primarily by a delay in diagnosis and the presence of associated injuries.

## Competing interests

The authors declare that they have no competing interests.

## Authors' contributions

SO conceived the study, participated in its design and coordination, wrote, revised and submitted manuscript. RU participated in its design and coordination. KMC performed the statistical analysis and coordination. AA participated in its design and coordination. CO participated in its design and revised the manuscript. All authors have read and approved the final manuscript.
